# Some possibilities of correction and compensation in body posture regulation among children and youth with low degree scoliosis

**DOI:** 10.1186/1748-7161-7-S1-O64

**Published:** 2012-01-27

**Authors:** J Nowotny, A Brzek, O Nowotny-Czupryna, K Czupryna, M Plaszewski

**Affiliations:** 1 Institute of Physiotherapy in Higher School of Administration in Bielsko-Biala, Katowice, Poland; 2 Medical University of Silesia in Katowice, Poland

## Background

Postural alignments, secondary curves of spine and tendency to unequal body weight distribution are the compensatory mechanisms in scoliosis, eventually leading to disturbances in the regulation of body posture. The pathological pattern of incorrect posture, evokes a vicious circle of causes and effects, which probably includes alterations in body weight distribution to both feet [1-4].

## Objective

To examine the role of equal weight loading of both feet in posture regulation among children and adolescents with low-degree scoliosis.

## Materials and methods

A total of 115 participants, aged 7-19 years, were divided into three groups: low degree scoliosis (10-26° Cobb; n = 56), scoliotic posture (5-9°; n = 29), and without lateral spine curvature (n = 30). Three measurements of body arrangement and the weight distribution on feet were simultaneously taken using the photogrammetry and the podographic platform: in a free standing position, while attempt to correct body arrangement and with equal loading of both feet.

## Results

Unequal weight distribution was observed in free standing position in patients with scoliosis. Attempts to correct body arrangement worsened existing disproportion, especially in the left-side curvatures. Equal feet loading lead to the body disarrangement, even among non – scoliotic subjects.

## Conclusions

In subjects with low degree scoliosis the compensatory changes in the spatial arrangement of the body are usually accompanied by asymmetric distribution of foot pressure and the active attempt to correct the curvature enhances this asymmetry. Attempts to maintain symmetrical distribution of body weight result in significant deterioration of the posturometric parameters.
